# Intra-abdominal Splenosis Mimicking Metastatic Disease During Evaluation for Bacteremic Sepsis

**DOI:** 10.7759/cureus.104762

**Published:** 2026-03-06

**Authors:** Kian Memari, Daniela P Escobar, Sara Ali, Lissette P Lazo, Shane Williams, Peter Cohen

**Affiliations:** 1 Family Medicine, Palmetto General Hospital, Hialeah, USA; 2 Medicine, Nova Southeastern University Dr. Kiran C. Patel College of Osteopathic Medicine, Clearwater, USA; 3 Family Medicine, Nova Southeastern University Dr. Kiran C. Patel College of Osteopathic Medicine, Fort Lauderdale, USA

**Keywords:** peritoneal nodules, post-splenectomy complications, sepsis, splenosis, stroke mimics of malignancy, technetium-99m-labeled heat-damaged red blood cells

## Abstract

Splenosis is an uncommon sequela of traumatic splenic rupture or splenectomy, resulting from the heterotopic autotransplantation of viable splenic tissue. These ectopic implants most commonly occur within the peritoneal cavity and often remain clinically silent for decades. On cross-sectional imaging, however, splenosis may closely resemble metastatic malignancy, lymphoproliferative disorders, or peritoneal carcinomatosis, creating diagnostic uncertainty and frequently prompting extensive oncologic evaluation.

We report the case of a 60-year-old man with a remote history of traumatic splenectomy who presented with bacteremic sepsis and was incidentally found to have multiple intra-abdominal soft-tissue nodules on computed tomography. The radiographic appearance raised concern for disseminated malignancy. Blood and urine cultures grew *Klebsiella aerogenes*, and the patient improved with targeted antimicrobial therapy. Given the patient's surgical history and prior awareness of residual splenic implants, intra-abdominal splenosis was favored over malignancy. Mild elevations in tumor markers were interpreted as reactive changes in the setting of acute systemic infection. Definitive diagnosis was established using technetium-99m-labeled heat-damaged red blood cell scintigraphy.

This case emphasizes the importance of integrating clinical history, imaging findings, and functional nuclear medicine studies when evaluating disseminated intra-abdominal lesions. Recognition of splenosis as a benign mimic of malignancy can prevent unnecessary invasive procedures and inappropriate oncologic workup, particularly in acutely ill patients.

## Introduction

Splenosis refers to the heterotopic implantation and revascularization of viable splenic tissue following traumatic splenic rupture or splenectomy. During splenic injury, fragments of splenic pulp may disperse throughout the peritoneal cavity and subsequently implant on serosal surfaces, where they establish an independent blood supply from surrounding tissues rather than the splenic artery [1]. The reported incidence of splenosis following traumatic splenic injury ranges from 25% to 65%, although most cases remain clinically silent [2]. 

It is important to distinguish splenosis from a congenital accessory spleen (splenule). Accessory spleens are congenital, structurally organized, and typically located near the splenic hilum, whereas splenosis is an acquired process characterized by irregular, unencapsulated implants lacking normal splenic architecture.

Despite its benign nature, splenosis presents a significant diagnostic challenge. On computed tomography (CT) and magnetic resonance imaging (MRI), splenic implants may appear as well-circumscribed, homogeneously enhancing soft-tissue nodules, closely resembling metastatic disease, lymphoma, or peritoneal carcinomatosis. This diagnostic pitfall is particularly consequential in patients undergoing evaluation for malignancy or systemic infection, where inflammatory marker elevation may further confound interpretation [3].

Functional nuclear medicine imaging using technetium-99m-labeled heat-damaged red blood cells (Tc-99m HDRBC) is considered the diagnostic gold standard due to its high sensitivity and specificity for splenic tissue. Accurate recognition of splenosis is essential to prevent unnecessary biopsies, surgical exploration, psychological distress, and inappropriate oncologic management [4].

We present a case of intra-abdominal splenosis incidentally identified during evaluation for bacteremic sepsis, highlighting how acute inflammatory states and tumor marker elevation can amplify concern for malignancy and emphasizing the critical role of functional imaging in establishing the correct diagnosis.

## Case presentation

A 60-year-old man with a history of childhood traumatic splenectomy presented to the emergency department with acute-onset fever, malaise, diffuse abdominal pain, severe headache, nausea, and non-bloody, non-bilious emesis. Symptoms began approximately 12 hours prior to presentation. On arrival, his temperature was 38.9°C and his heart rate was 112 beats per minute, meeting the criteria for systemic inflammatory response syndrome.

Initial laboratory evaluation revealed leukocytosis with neutrophilic predominance and left shift, elevated inflammatory markers, and mild transaminitis (Table 1). Given severe abdominal pain and concern for intra-abdominal pathology, a CT of the abdomen and pelvis was performed with and without intravenous contrast.

**Table 1:** Detailed laboratory investigations µL: microliter; mm³: cubic millimeter; gm/dL: grams per deciliter; fL: femtoliter; pg: picogram; mmol/L: millimoles per liter; mg/dL: milligrams per deciliter; mcg/dL: micrograms per deciliter; ng/mL: nanograms per milliliter; mIU/mL: milli-international units per milliliter; U/L: units per liter; mg/L: milligrams per liter; mL/min/1.73 m²: milliliters per minute per 1.73 square meters of body surface area; WNL: within normal limits; AST: aspartate aminotransferase; ALT: alanine aminotransferase; TSH: thyroid-stimulating hormone; Ag: antigen

Parameter (unit)	Patient's value	Normal reference range	Clinical note/interpretation
White blood cell (µL)	22.1	5.0-11.0x10^3^	-
Red blood cell (mm^3^)	5.17	4.70-6.10x10^6^	WNL
Hemoglobin (gm/dL)	15.2	14.0-18.0	WNL
Hematocrit (%)	44.7	42.0-52.0	WNL
Mean corpuscular volume (fL)	87	80-94	WNL
Mean corpuscular hemoglobin (pg)	29.4	27.0-31.0	WNL
Mean corpuscular hemoglobin concentration (gm/dL)	34.0	33.0-37.0	WNL
Red cell distribution width: coefficient of variation (%)	13.6	11.5-14.5	WNL
Platelet count (µL)	323	130-140x10^3^	WNL
Sodium (mmol/L)	137	137-145	WNL
Potassium (mmol/L)	4.5	3.4-5.0	WNL
Chloride (mmol/L)	99	98-107	WNL
Carbon dioxide (mmol/L)	29	22-30	WNL
Anion gap (mmol/L)	9	10-20	-
Blood urea nitrogen (mg/dL)	17	9-20	WNL
Creatinine (mg/dL)	0.90	0.66-1.25	WNL
Estimated glomerular filtration rate (mL/min/1.73 m^2^)	98	≥90	WNL
Glucose (mg/dL)	250	74-106	-
Lactic acid (mmol/L)	1.5	0.7-2.0	WNL
Calcium (mg/dL)	8.8	8.4-10.2	WNL
Phosphorus (mg/dL)	3.4	2.5-4.5	WNL
Magnesium (mg/dL)	1.80	1.60-2.30	WNL
Total bilirubin (mcg/dL)	2.30	0.20-1.30	-
Direct bilirubin (mg/dL)	0.0	0.0-0.3	WNL
Indirect bilirubin (mg/dL)	2.3	0.0-0.1	-
AST (U/L)	123	17-59	-
ALT (U/L)	106	21-72	-
Alkaline phosphatase (U/L)	149	38-126	-
Lactate dehydrogenase (U/L)	281	120-246	-
Troponin I (ng/mL)	<0.012	0.012-0.034	-
C-reactive protein (mg/L)	43.6	<10.0	-
Lipase (U/L)	83	23-300	WNL
Carcinoembryonic Ag (ng/mL)	4.5	0.0-3.0	-
Prostate-specific Ag (ng/mL)	4.17	0.00-4.00	-
Procalcitonin (ng/mL)	3.85	<0.50	-
TSH (reflex) (mIU/mL)	0.849	0.465-4.680	WNL

**Figure 1:**
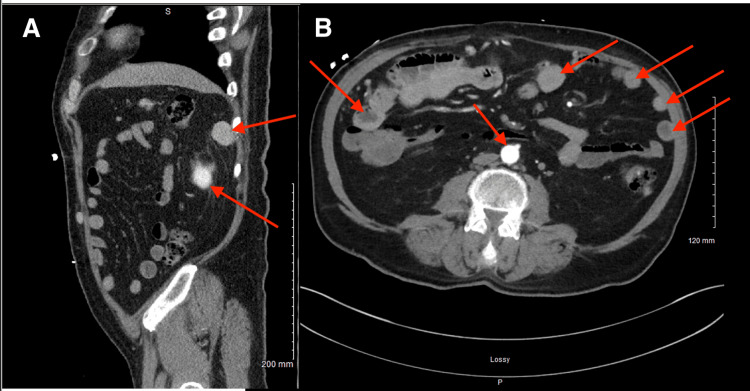
CT of the abdomen and pelvis: sagittal contrast-enhanced (A) and axial non-contrast (B) images (A) Sagittal contrast-enhanced CT of the abdomen and pelvis demonstrates several circumscribed, homogeneously enhancing masses scattered throughout the abdomen, including the left upper quadrant, anterior peritoneum, and lower pelvis (red arrows). These findings are indeterminate and may represent splenosis; however, alternative etiologies such as enlarged lymph nodes or peritoneal metastases cannot be excluded. Additional enhancing hepatic lesions may represent a similar etiology to the intra-abdominal masses. (B) Axial non-contrast CT of the abdomen and pelvis demonstrates multiple soft-tissue nodular lesions within the pelvis and peritoneum (red arrows), raising concern for splenosis versus peritoneal metastases. Tissue biopsy is recommended for definitive diagnosis. Soft-tissue lesions are also noted along the right common iliac and right internal iliac nodal stations. CT: computed tomography

CT imaging demonstrated multiple well-circumscribed, homogeneously enhancing soft-tissue nodules scattered throughout the peritoneum and pelvis, ranging from 0.8 cm to 2.5 cm, raising concern for metastatic disease or peritoneal carcinomatosis (Figure 1). 

Empiric intravenous ceftriaxone was initiated, later broadened to piperacillin-tazobactam with adjunctive tobramycin due to persistent sepsis. Blood and urine cultures grew *Klebsiella aerogenes*. Antibiotic therapy was de-escalated to intravenous cefepime based on susceptibilities, followed by oral levofloxacin 750 mg once daily for 10 days upon discharge.

Tumor markers were mildly elevated, including carcinoembryonic antigen (4.5 ng/mL) and prostate-specific antigen (4.17 ng/mL), interpreted as nonspecific elevations in the setting of acute systemic inflammation.

**Figure 2:**
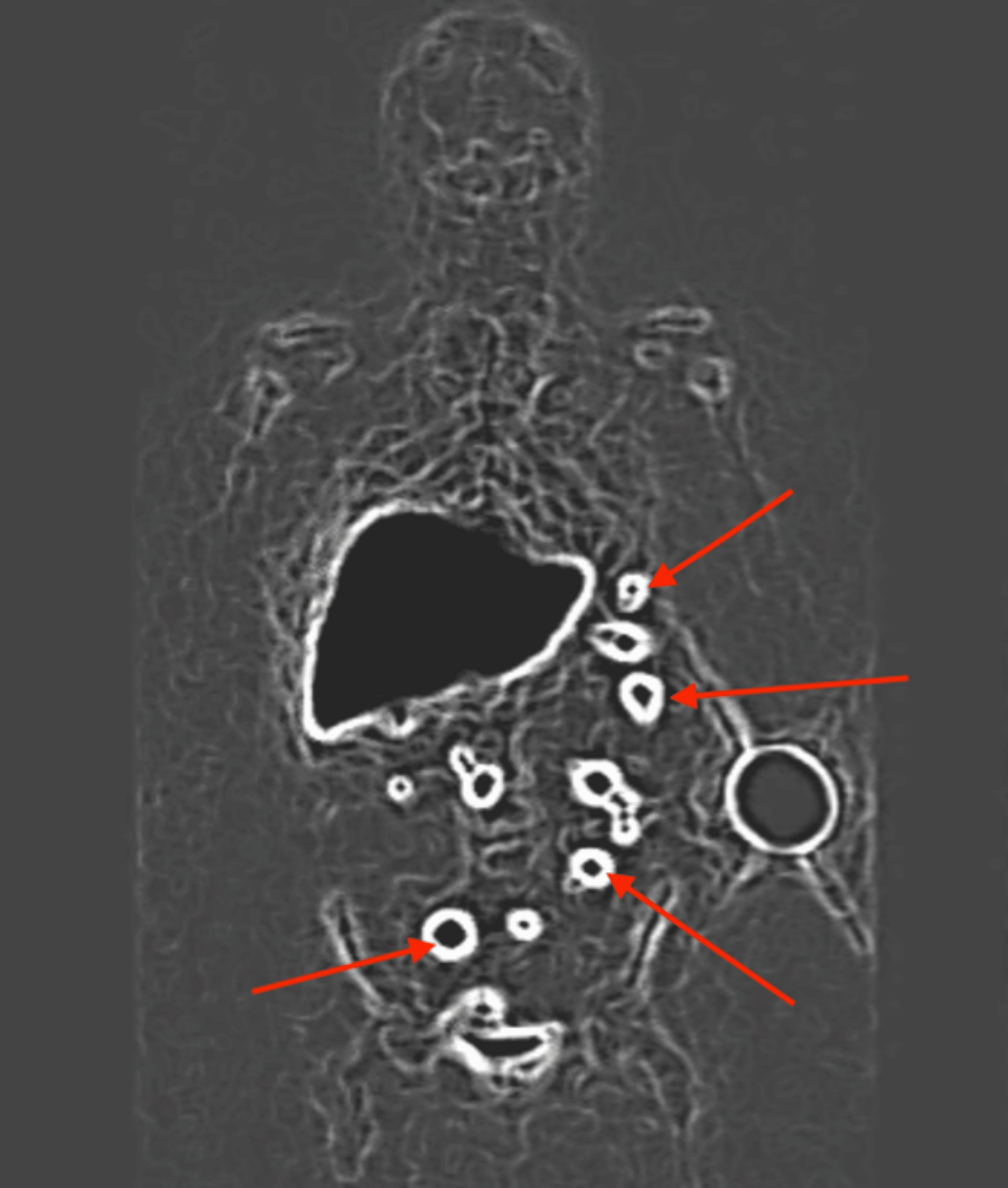
Tc-99m HDRBC scintigraphy Scintigraphic imaging demonstrates radiotracer uptake within multiple peritoneal nodules (red arrows), with activity equivalent to native splenic tissue, confirming the diagnosis of intra-abdominal splenosis. Tc-99m HDRBC: technetium-99m-labeled heat-damaged red blood cell

Given the patient's known history of traumatic splenectomy and prior counseling regarding possible residual splenic implants, intra-abdominal splenosis was favored. Tc-99m HDRBC scintigraphy demonstrated radiotracer uptake within the peritoneal nodules equivalent to native splenic tissue, confirming the diagnosis (Figure 2).

The patient's fever, leukocytosis, C-reactive protein (CRP), and procalcitonin improved with antimicrobial therapy. He was discharged in stable condition with outpatient follow-up.

## Discussion

Splenosis results from the mechanical dissemination of splenic tissue following the traumatic rupture or surgical removal of the spleen. Implanted splenic tissue revascularizes and may retain immunologic function, which may partially mitigate, but not eliminate, the risk of overwhelming post-splenectomy infection [1].

This case highlights splenosis as a major diagnostic challenge, particularly in acutely ill patients. In the setting of bacteremic sepsis, imaging findings suggestive of disseminated malignancy and concurrent tumor marker elevation substantially increased diagnostic uncertainty. Importantly, splenosis is increasingly recognized as a cause of false-positive findings on molecular imaging, including 68Ga-DOTATATE positron emission tomography/CT (PET/CT), where it may prompt unnecessary treatment escalation. Correlation with Tc-99m HDRBC scintigraphy is therefore recommended when splenosis is suspected in patients with a history of abdominal trauma [5].

CT and MRI lack sufficient specificity to reliably differentiate splenosis from malignancy. Tc-99m HDRBC scintigraphy remains the gold standard due to selective splenic uptake, as demonstrated in this case [3].

Failure to recognize splenosis can result in unnecessary invasive diagnostic procedures and inappropriate oncologic workup. Integration of surgical history, imaging characteristics, laboratory trends, and functional imaging is essential to avoid diagnostic error.

## Conclusions

Intra-abdominal splenosis is a benign but frequently underrecognized condition that may closely mimic metastatic disease on conventional imaging. This case demonstrates how splenosis may be incidentally identified during evaluation for acute bacteremic sepsis and emphasizes the importance of historical context and functional imaging in establishing the correct diagnosis. Awareness of splenosis as a malignancy mimic can prevent unnecessary invasive procedures and inappropriate oncologic management.
